# *Moringa oleifera* Leaf Extracts as Multifunctional Ingredients for “Natural and Organic” Sunscreens and Photoprotective Preparations

**DOI:** 10.3390/molecules23030664

**Published:** 2018-03-15

**Authors:** Anna Baldisserotto, Piergiacomo Buso, Matteo Radice, Valeria Dissette, Ilaria Lampronti, Roberto Gambari, Stefano Manfredini, Silvia Vertuani

**Affiliations:** 1 Department of Life Sciences and Biotechnology, Master Course in Cosmetic Science and Technology, University of Ferrara, Via L. Borsari 46, 44121 Ferrara, Italy; anna.baldisserotto@unife.it (A.B.); piergiacomo.buso@student.unife.it (P.B.); valeria.dissette@unife.it (V.D.); ilaria.lampronti@unife.it (I.L.); roberto.gambari@unife.it (R.G.); vrs@unife.it (S.V.); 2 Universidad Estatal Amazónica (Km 2 ½ Via Napo (Paso Lateral)), Puyo, Pastaza EC160150, Ecuador; matteo.radice@gmail.com; 3 Ambrosialab Srl, Via Mortara 171, 44121 Ferrara, Italy

**Keywords:** *Moringa oleifera*, natural sunscreen, sustainable sources, SPF determination, dermo-cosmetic safety, anti-proliferative activity

## Abstract

*Moringa oleifera* has gained increasing popularity as a food supplement but not in the pharmaceutical and cosmetic area. The aim of this study was the preparation, characterization, and evaluation of extracts from the leaves of *Moringa oleifera* as a herbal sun care phytocomplex. Three different extracts of *Moringa oleifera* leaves, from Senegal, have been prepared and chemically characterized in the phenolic fraction by HPLC-DAD and Folin–Ciocalteu test. To explore photoprotective properties, an extensive evaluation of UV filtering, antioxidant (DPPH, FRAP, ORAC, PCL), and anti-hyperproliferative (human melanoma Colo38 cells) capacities have been conducted. Furthermore, a formulation study regarding cosmetic prototypes has been carried out in order to determine the Sun Protection Factor (SPF), which was assessed in vitro. The extracts were demonstrated to confer significant values of protection, with an SPF 2, that corresponds to a 50% protection against UV-B rays, at concentrations as low as 2% to 4%. Finally, the evaluation on potential irritation of the finished formulations was conducted by Patch Test and no significant irritant potential was observed. These evidence enlarged the already significant number of activities and potential uses of this plant, which is well-known for its importance in the medicinal and nutritional fields.

## 1. Introduction

Daily photoprotection is becoming a very important health issue in view of the ever-increasing number of skin neoplastic diseases, but customers are also concerned by the level of risk that may be held by synthetic chemical sun filters. This increases the demand for natural products that are very rarely supported by sound investigations and may induce higher risks than traditional sun filters. Plant extracts contain many active compounds that may work as radicals scavengers being thus able to protect skin matrix from enzymatic degradation. Furthermore, phytochemicals as flavonoids and phenolic compounds possess a structure that allows them to absorb protons and rapidly return to ground state just like chemical UV filters. The association in sunscreen formulations of UV filters with plant extracts can be a valuable natural strategy to extend skin protection against UV radiation damage. In this context, many plant species have been investigated for their potential uses in terms of solar radiation protection [1,2,3], although not always through a systematic scientific approach. *Moringa oleifera* (Moringaceae) is a plant native to sub-Himalayan tracts of India, Pakistan, Bangladesh, and Afghanistan that can grow in tropical and subtropical climatic areas. It is mainly cultivated in the Middle East Africa and Southern Asia for multiple purposes. It is commonly known as “drumstick tree” or “horseradish tree” [4,5]. A plethora of traditional uses and ethnomedical practices are reported and confirm the medicinal and nutritional value of this plant. Cancer treatment, anti-inflammatory and anti-Buruli ulcer activity, fertility problems, and lifestyle-related diseases, such as high blood pressure and diabetes mellitus, have been mentioned as the main traditional uses in several tropical countries. [6,7,8,9,10,11,12,13]. In recent years, many ethno-pharmaceutical studies have appeared in the scientific literature extending the range of possible applications: we can find reports regarding the antioxidant, antimicrobial, anti-inflammatory, anti-proliferative, antispasmodic, antiulcer, hypocholesterolemic, and hypoglycemic activities of different parts of *M. oleifera* [5,14]. Due to these characteristics, *Moringa oleifera* can be considered as one of the most useful plants of the world as concerns its potential uses, and not surprisingly it is termed the “Tree of life” [15].

Sun light is composed of about 40% visible light (VIS), 50% infrared light (IR), and 10% ultraviolet light (UV). As concerns the biological effects of solar radiation exposure, ultraviolet radiation is the most important fraction of the electronic spectrum. It can be divided into UVC (200–280 nm), UVB (280–315 nm), and UVA (315–400 nm). UVC is almost completely filtered by atmospheric oxygen and ozone; UVB and UVA are partially filtered [16]. UV radiation is necessary for the production of Vitamin D3 in the skin, and this activity mediates the major part of the positive effects of solar radiation that also has therapeutic effects on skin diseases, such as psoriasis and eczema. Vitamin D3 is necessary to the intestinal absorption of calcium and phosphorus; a deficiency can cause osteoporosis in adults and growth retardation and skeletal deformities in children [16]. On the other hand, the interaction of UV radiation with various chromophores in the skin, such as DNA, RNA, proteins, lipids, and aromatic amino acids, represents the basis of UV-mediated negative biological activities [17]. More specifically, UVB radiation is responsible for UVB-induced erythema, which is the most well-known acute negative effect that occurs a few hours after exposure to UV rays. This radiation has strong carcinogenic effects because it causes direct damage to DNA and RNA. UVA radiation induces chronic damages that are the main factor responsible for skin ageing; it leads to an excessive degradation of collagen fibres and to an inhibition of the replacing of collagen and hyaluronic acid [16]. UV radiation is also responsible for reduction of the activity of endogenous enzymatic and non-enzymatic antioxidant systems in human skin. In the field of solar protection, the evaluation of the anti-proliferative activity of new compounds and raw materials is a matter of overriding importance; this activity poses a leading position in the list of desirable biological activities for a UV-filtering compound or raw material.

The increasing of scientific reports concerning the photoprotective activity of natural products observed during the last decade has not been followed by the official approval of any natural sun-filter; nonetheless, the association of approved traditional (chemical or physical) sun-filters with herbal derivatives represents an affirmed and common trend in the cosmetic field, especially those making natural and green claims, which consumers perceive to be the most safe. The reason for this can be found in reports of interesting values of UV absorption, or in other types of biological activities of vegetal extracts and raw materials that have equal importance in the prevention of UV-exposure-connected damages. Sun protection represents a complex issue because it involves various biological activities and factors that modulate the efficacy of a sunscreen. They can be summarized in six main categories: protection against UVB/UVA radiation, the scavenging activity of anti-radical and reactive oxygen species, antimutagenic activity, anti-proliferative properties, booster effect, safety, and stability. Taking this into account, and in view of our interest in the field [18,19], this work was intended to evaluate extracts of *Moringa oleifera* for their potential uses in solar protection considering complementary biological activities that can be useful in this field, and conducting the chemical characterization with particular attention to compounds that are known to play a central role in photoprotection.

## 2. Results

For the current study, three different extracts of *Moringa oleifera* leaves were prepared and analyzed: hydroalcoholic, methanolic, and water extract.

All of the extracts were evaluated for their antioxidant activity in vitro by DPPH, PCL, FRAP, and ORAC assays. Results are summarized in Table 1 and are expressed in IC_50_ values (µg/mL) for DPPH and as µmol TE/g for the PCL, FRAP, and ORAC tests.

The study *Moringa oleifera* leaf extracts were demonstrated to possess interesting antioxidant activity. Not all of the tested extracts performed equivalently; this was due to various reasons, such as the different interactions with the radicals exploited in the tests and the heterogeneous nature of a vegetal phytocomplex. This activity can be generated mostly by the presence of polyphenols, which are known to scavenge free radicals [20]. Antioxidant activity plays an important role in photoprotection because of its usefulness against UV-exposure-mediated oxidative stress and because of its special benefits for the skin.

The DPPH values indicate modest activity if compared to other plant extracts known for their interesting antioxidant profile. For example, Chandra et al. report the antioxidant activity of extracts obtained by some Indian medicinal plants [21]. Among these, those with the most significant antioxidant profile were the methanolic extract of *Trimufetta rotundifoglia* 17 μg/mL and *Paltophorum ferrugineum* 20.5 μg/mL. In the case of *Moringa oleifera*, the best results were obtained with the hydroalcoholic and the aqueous extracts.

The ORAC values indicate good antioxidant activity if compared with other plant extracts known for their high activity. For example, other authors report the antioxidant activity of cinnamon *Cinnamomum verum* aqueous extract (8515 µmol Trolox equivalent per gram, µmol TE/g), pine *Pinus maritima* aqueous extract (7727 µmol TE/g), Cabreuva *Myrocarpus fastigiatus* aqueous extract (5422 µmol TE/g), *Mate Ilex paraguariensis* aqueous extract (5092 µmol TE/g), and oak *Quercus robur* aqueous extract (3850 µmol TE/g) [22]. PCL values of the analysed extracts showed a significant antioxidant activity as compared with results previously obtained by us on other vegetal extracts [23].

The FRAP test highlighted an interesting antioxidant profile. The results are in line with those of other works. For example, Fernandes et al. reported comparable data for other plants known for their antioxidant activity: *Origanum vulgare* (472.32 ± 15.96 μmol Trolox/g), *Origanum majorana* (463.96 ± 28.49 μmol Trolox/g), *Rosmarinus officinalis* (361.57 ± 33.72 μmol Trolox/g), and *Melissa officinalis* (464.83 ± 25.96 μmol Trolox/g) [24].

The total polyphenol content of the different types of extracts is presented in Table 2.

### 2.1. Quantitative Estimation of Polyphenols

A freeze-dried infusion, a hydroalcoholic extract, and a methanolic extract of *Moringa oleifera* have been characterized and titled by HPLC in selected components. A quantitative estimation of the polyphenols is given in Table 3 according to the peak area. The profile of the three extracts differs in their qualitative composition (Quercetin was detected only in the hydroalcoholic extract), while there were no large quantitative differences.

As supported by a large body of literature, the selected compounds possess biological activities that can be useful in solar protection, and confirm the great potentialities of *Moringa oleifera* leaf extracts as an active ingredient in solar products.

Ellagic acid, as reported by Hseu et al. (2012), suppresses UVA-induced oxidative stress and may be useful in the treatment of UVA-induced skin damage, photoaging, and skin cancers. Moreover, Young Bae et al. (2009) mentioned that ellagic acid could prevent collagen destruction and reduce the inflammatory responses caused by UVB exposure on UVB-irradiated human skin cells and hairless mice. These remarkable findings suggest that ellagic acid is a valuable ingredient for the formulation of skin care products [25,26].

Chlorogenic acid presents anti-proliferative activity as reported by Kang et al. (2013). It enhances the expression of immune factors, such as IL-2R and IFN-𝛾, and consequently promotes the activation and proliferation of T cells, macrophages, and NK cells, thus enhancing their surveillance and killing abilities [27]. Fukushima et al. (2015) mentioned that chlorogenic acid could protect human skin from photoaging due to its antioxidant properties [28].

Ferulic acid has been demonstrated to have strong antioxidant activity and, as reported by several authors, can be used in solar products as an active ingredient. This compound has a protective role against ultraviolet-induced erythema [29,30,31].

Rutin (quercetin-3-*O*-rutinoside) is a flavonoid with remarkable antioxidant activity. It can effectively boost the activity of the widely used UV filter benzophenone-3 [32]. There is also a preliminary study regarding the development of a lipidic colloidal system that can enhance rutin’s antioxidant and photoprotective activities [33].

Quercetin is a flavonoid with UV photoprotective properties. Choquenet et al. have reported that quercetin, incorporated in oil-in-water emulsions at a concentration of 10% (w/w), confers interesting in vitro sun protection factor (SPF) values to a formulation. A non-negligible level of photoprotection in the UVA range is also reported [34].

### 2.2. Formulation Approach

Because even the high potency of an extract is meaningless if not expressed also within a formulation, we investigated the skin tolerability and the SPF in finished dermo-cosmetic formulations. An extensive formulation study has been conducted in order to find suitable concentrations of extracts. At the end, the three dried extracts (INCI: *Moringa oleifera* Leaf Extract) were included at two different concentrations (2% and 4%) in the water phase of a standard oil in water (O/W) emulsion, suitable for cosmetic use, to test the UV filtering capacity and tolerability.

### 2.3. Skin Tolerability

For a preliminary assessment of skin tolerability of the products containing the *Moringa oleifera* extract, the O/W emulsion was subjected to a patch test in occlusion on 20 volunteers. The participants were recruited among the students of the Department of Life Science and Biotechnology, University of Ferrara (Italy). Informed consent was obtained from all participants, and the study was approved by the ethical committee of the University of Ferrara (Approval by the Ethical Committee of the University of Ferrara (N.1/96)). This study assesses the potential skin reactivity (skin erythema and oedema reactions) that may occur after applying a cosmetic product under drastic conditions for 48 hours in occlusion to evaluate whether the topical product is safe for consumer use. The finch chambers containing the various formulations to be tested, enclosed in aluminum circular cells, were applied to the forearm flying area or to the voluptuous area of the volunteers. Test result readings were performed at two different times: the first one after 15 minutes of application of the patch and the second after 24 hours by a dermatologist. For the full definition of this analysis, the average of all the results obtained from the individual volunteers is made. This study has been carried out in compliance with the most recent recommendations of the Helsinki Declaration (64th WMA General Assembly, Fortaleza, Brazil, October 2013) and has followed the “Guidelines for the Assessment of Skin Tolerance of Potentially irritant Cosmetic ingredients”, COLIPA, 1997.

Epicutaneous patch testing was performed by means of adhesive strips for patch tests in a sufficient amount to fill one test disk (approximately 0.07–0.1 mL) before occluded application to the skin of the forearm of each volunteer [35].

For all 20 volunteers (data not shown), the dermatological product test resulted in a mean index of irritation of:

- **0**, **10** (zero, ten) 15 min after the removal of the Finn Chamber;
- **0**, **10** (zero, ten) 24 h after the removal of the Finn Chamber.

On the basis of these Minimum Irritation Index (MII) values it is clear that, according to the evaluation scale used, the product can be classified as NOT IRRITATING (MII < 0.5) if applied to human skin.

### 2.4. In Vitro SPF Determination

The in vitro SPF evaluation demonstrated that all three plant extracts have an interesting UV filtering profile if included in formulations compatible with cosmetic use (Table 4).

The SPF values of the tested formulations, considering concentrations of the *Moringa oleifera* leaf extracts ranging from 2% to 4%, were compatible with SPF 2, which means that about 50% of the UVB radiation was filtered by the finished product. This represented an encouraging performance in the field of herbal extracts, which are usually sources of heterogeneous phytocomplexes that also contribute to photoprotection by other complementary mechanisms (i.e., antioxidant, lenitive).

All of the tested formulations were photostable, as no significant variations in the SPF values were shown after irradiation had been conducted, as prescribed by the ISO 24443:2012 procedure, with a solar simulator device (Suntest CPSþ; Atlas, Linsengericht, Germany) (data not shown). Despite the relatively low SPF values, the UVA protection factor (UVAPF0) appeared to be of great interest. In fact, the Critical Lambda values of all of the formulations were greater than 370 nm and the UVA/UVB Ratio was close to 1, which is indicative of a broad spectrum filtering activity. The complexity of the mixtures of compounds present in a vegetal raw material is always an obstacle to the identification of the molecules contributing most to the studied activity. Further studies are needed to clarify which of the characterized compounds or group of compounds is more effective as concerns the filtering activity and how geographic location and harvest time affect the performance of the various types of extracts of *Moringa oleifera*.

### 2.5. Anti-Proliferative Effects of Extracts from Moringa oleifera on Human Melanoma Colo38 Cell Line

**Figure 1:**
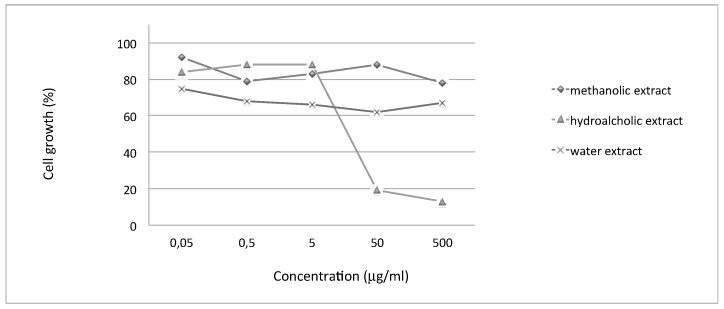
Effects of methanolic (rhombus symbols), hydroalcoholic (triangle symbols), and water (asterisk symbols) extracts on human colo38 cell proliferation described in a representative experiment.

The anti-proliferative effects of *Moringa oleifera* extracts were evaluated on human melanoma Colo38 cells. In our experiments, cells were seeded at 40,000 cells/mL and cultured in the presence of increasing concentrations of *Moringa oleifera* extracts (0.05, 0.5, 5, 50, and 500 µg/mL). The cell count was performed after 48 h and 72 h of cell culture, when cells are in the log phase of growth. Table 5 and Figure 1 indicate the anti-proliferative effects (IC_50_ values) of the tested extracts (the experiments were repeated at least 3 times under the same conditions) underlying the biological activity of the hydroalcoholic extract. The IC_50_ value (Inhibitory Concentration 50%) is the concentration of drug that is required for 50% cell growth inhibition.

The biological activity of several extracts from *Moringa oleifera* were studied on human tumor cell lines and data were published in the literature, starting from a first publication [36] in which ethanolic extracts, derived from roots, were assayed on different cell cultures (lymphoma Raji, T lymphocyte Jurkat, eritroleukemia K562, and HEL human cells) and demonstrated anti-proliferative activity at 5–50 μg/mL concentration. Most recently, a crude aqueous leaf extract of *Moringa oleifera* was demonstrated to be active in inhibiting the cell growth of cancerous human alveolar epithelial A549 cells [37]. Also, extracts derived from seeds were studied for their anti-proliferative effect on breast cancer MCF7 [38]. Finally, a *Moringa oleifera* seed lectin was purified and its anti-proliferative activity was studied against Ehrlich ascites carcinoma (EAC) cells, demonstrating that the cell growth inhibition was due to the induction of apoptosis [39]. To the best of our knowledge, this is the first investigation on possible *Moringa oleifera* anti-proliferative effects on melanoma colo38 cells. In the present study, we have also observed that the hydroalcoholic extract obtained from the leaves of *Moringa oleifera* was the only one that possesses interesting anti-proliferative activity on this cell line associated with photoprotective and antioxidant activity.

## 3. Discussion

The increasing number of recent studies confirms that natural products have become increasingly important for future trends in sunscreen formulation and skin photoprotection [18,40]. In order to identify new natural and effective herbal sun care products from renewable sources, the above-mentioned extracts from *Moringa oleifera* leaves showed promising results, which justify further studies in this field. All three extracts, due to their polyphenols content, expressed interesting antioxidant activity and UV filter properties. Hydroalcoholic extract adds also an important anti-proliferative effect, which completes a remarkable set of key factors for skin photo-aging and skin cancer prevention. The presence of quercetin in the hydroalcoholic extract can partially explain this finding, but further studies are necessary to evaluate the synergistic effects of the other components of the phytocomplex. All tested emulsions presented good photostability, an interesting UVA/UVB protection factor, and broad spectrum filtering activity. Furthermore, no significant irritant potential has been reported from a Patch Test. Taking all of this into account, *Moringa oleifera* seems to be a good candidate species for sun care and skin-aging protection because its extracts, especially the hydroalcoholic one, showed a “cluster” of useful biological activities which synergistically work in order to prevent or to reduce UV-induced damage. In closing, the promising in vitro results should be extended at an in vivo model in order to complete the research. 

## 4. Materials and Methods

### 4.1. Plant Material and Extraction Methods

*Moringa oleifera* leaves were harvested in August 2016 (Lot number: 34FE16). After collecting, the leaves were dried by Baobab Fruit Company Senegal SARL (BFCS, Touba Peycouk BP 826, Thies, Senegal). Dried sample was packaged and sent to our laboratory. Upon arrival, dried leaves were ground to a fine powder with a mortar and stored at −80 °C. The extraction processes have been conducted as previously described by Vongsak 2013 [41] and Nouman 2016 [42] with some modifications.

**Hydroalcoholic extract:** About 10 g of powder was blended with 200 mL of hydroalcoholic solution (ethanol: distilled water, 70:30) at room temperature for 60 min under magnetic stirring. The residue was filtered and concentrated in vacuum to afford the desired hydroalcoholic dried extract.

**Methanolic extract:** To obtain methanolic dried extract, about 5 g of powder was blended with 100 mL of Methanol and subjected to two sonication cycles (40°, 60 min, 80%) with subsequent centrifugation. The supernatant was concentrated under vacuum to obtain the desired product.

**Decoction:** 10 g of powder was blended with 150 mL of distilled water under magnetic stirring at 100 °C for 45 min. The decoction solution was filtered and lyophilized to obtain the dried water extracts.

### 4.2. In Vitro Antioxidant Activities

#### 4.2.1. DPPH Radical Scavenging

The DPPH radical scavenging activity of the different extracts of *M. oleifera* leaves was determined according to a described procedure [43]. This assay is used to determine the antioxidant capacity in a brief time and it is ideal for phenolic compounds.

It is possible to evaluate the ability of an antioxidant to donate hydrogen to convert the free stable radical DPPH in 1,1-diphenyl-2-picrylhydrazyl by measuring the absorbance decrease to 517 nm of the solution containing the tested product that changes colour from deep-violet to light-yellow after the reaction with the radical. The percentage was calculated using the following equation:

DPPH radical-scavenging capacity (%) = [1 − (A1 − A2)/A0] × 100%

where A_0_ was the absorbance of the control (without sample), A_1_ was the absorbance in the presence of the sample, and A_2_ was the absorbance without DPPH. The IC_50_ values, defined as the amount of antioxidant necessary to decrease the initial DPPH˙ concentration by 50%, were calculated from the results. To a DPPH methanolic solution (1.5 mL) was added 0.750 mL of extract solution (methanol) at different concentrations (from 30 to 600 µg/mL), and the absorbance was measured by a UV-Vis spectrophotometer (DU^®^ 530 LifeScience, Beckman Coulter^TM^). IC_50_ values were expressed as µg/mL and determined by regression analysis of the results obtained at different concentrations of the sample.

#### 4.2.2. PCL (Photochemiluminescence)

The PCL assay is based on Popov and Lewin’s method [44] and measures the antioxidant activity of a sample against superoxide anion radicals with a Photochem^®^ apparatus (Analytik Jena, Leipzig, Germany). Radicals are generated from Luminol, a photo-sensitizer agent, as a result of exposure to UV light (Double Bore^®^ phosphorus lamp, output 351 nm, 3 mWatt/cm^2^). The antioxidant capacity was measured using the manufacturer’s ACL (Antioxidant Capacity of Liposoluble substance) kit.

The kinetic light emission curve, which exhibits no lag phase in ACL studies, was monitored for 180 s and expressed as micromoles of Trolox (standard) per gram of dry matter. The areas under the curves were calculated using the PCL soft control and analysis software.

The dried extracts were diluted in methanol prior to analysis. The antioxidant assay was carried out in triplicate for each sample, and 20 μL of the diluted extract (1:20, *v*/*v*) in HPLC-grade methanol were sufficient to correspond to the standard curve.

#### 4.2.3. ORAC (Oxygen Radical Absorbance Capacity)

The ORAC assay was performed based on the Hong procedure that was modified in a previous work [45] using a Fluoroskan FL^®^ ascent (Thermo Fisher Scientific, Inc., Waltham, MA, USA) with fluorescent filters (excitation wavelength: 485 nm; emission filter: 538 nm).

Fluorescein sodium salt (85 nM) was used as a target of free radical attack with 2,2′-azobis(2-amidinopropane) dihydrochloride (AAPH) as a peroxyl radical generator in the final assay mixture (0.2 mL total volume). A calibration curve of Trolox (standard control) was carried out with different concentrations (from 40 to 240 μM solutions). The tested dried extracts were dissolved in methanol and then diluted in PBS (Phosphate Buffer Solution pH 7.4). The fluorescence measurements were carried out at 37 °C and recorded at 5-min intervals up 30 min after the addition of AAPH. The ORAC values were calculated as the difference of the areas under the quenching curves of fluoresceine between the blank and the sample, and were expressed as µmol Trolox equivalents (TE) per gram of dried matter.

#### 4.2.4. FRAP (Ferric Reducing Antioxidant Power) Assay

We adopted the typical FRAP method by Benzie and Strain [46] that measures the ferric reducing ability of plasma, and it is based on the reduction of ferric ions (Fe^3+^) to ferrous ions (Fe^2+^) in the presence of TPTZ (2,4,6-tripyridyl-s-triazine) under acid conditions. The ferric-tripyridyltriazine (Fe(III)-TPTZ) complex, in the presence of an antioxidant, was reduced to the ferrous (Fe(II)) form and an intense blue color with an absorption maximum at 593 nm was observed. FRAP reagent was prepared immediately prior to the analysis by mixing 0.1 M acetate buffer pH 3.6, TPTZ 10 mmol/L in 40 mmol/HCl, and ferric chloride 20 mmol/L in ratio 10/1/1. To 0.1 mL of the diluted sample (or solvent when blank was performed) was added 1.9 mL of FRAP reagent. All of the samples were incubated in the dark at 37 °C for 10 min, and then the absorbance was measured at 593 nm using a UV-VIS spectrophotometer.

A calibration curve was prepared by using Trolox as standard. The parameter evaluated to calculate the antioxidant activity was the absorbance increase of the sample solution against the absorbance of the blank. The results were expressed as µmol Trolox equivalents (TE) per gram of dried matter.

### 4.3. Total Polyphenol Content

An adapted and optimized Folin–Ciocalteu method [47] was used to evaluate the polyphenol content of the extracts. A calibration curve was obtained using Gallic acid as standard at different concentrations (0, 50, 100, 150, 250, and 500 ppm). A mixture of water-diluted Folin–Ciocalteu reagent (1/15) (1.5 mL aliquot) and the extracts (20 µL) was incubated at room temperature for 5 min and then, after adding 300 µL of a solution of sodium carbonate (200 g/L), was incubated at room temperature in the dark for a further 90 min. Finally, the absorbance was measured at 765 nm using a UV-VIS spectrophotometer against a blank containing distilled water instead of the extracts. Results were expressed as equivalent to micrograms of Gallic acid equivalent (GAE) per milligram of a sample (µg of GAE/mg of dried extract).

### 4.4. Characterization of Polyphenols

#### 4.4.1. Preparation of Standard Solutions

Standard solutions of Chlorogenic acid, Rutin, Ellagic acid, Ferulic acid, and Quercetin were prepared in methanol and properly diluted with methanol to achieve the final concentration of 80, 40, 20, 10, 5, 2.5, 1.25, and 0.625 µg/mL.

#### 4.4.2. Sample Preparation

An accurately weighed aliquot (20 mg) of each extract was dissolved in 50% methanol (5 mL). Before injection, each solution was filtered through a 0.45 nylon membrane filter and analyzed in triplicate by HPLC.

#### 4.4.3. HPLC Apparatus and Chromatographic Conditions

HPLC analysis was performed using an Agilent 1100 Series HPLC System equipped with a G1315A DAD and with an Hydro RP18 Sinergi 80A column (4.6 × 250 mm, 4 μm) from Phenomenex. Separation was monitored with absorbance detection at 254 ± 8 nm. The elution was performed on a gradient solvent using water (0.01 M H_3_PO_4_) as solvent A and acetonitrile (0.01 M H_3_PO_4_) as solvent B. The ratios were as follows: 90:10 (A/B) to 80:20 (A/B) in 5 min, held for 5 min, 80:20 (A/B) to 20:80 (A/B) in 10 min, 20:80 (A/B) to 90:10 (A/B) in 2 min. The flow rate was 1.2 mL/min at room temperature. The injection volume for all samples and standards was 5 µL. The quantitative HPLC analysis was calculated, for each compound, according to its peak area.

### 4.5. Formulations

INCI: Aqua, Coco-caprylate, Cetearyl alcohol, Caprylic/capric triglyceride, Glyceryl stearate citrate, Tridecane, Undecane, Glyceryl caprylate, Benzyl alcohol, Xanthan gum, Benzoic acid, Propanediol, Phytic acid.

O/W emulsions were prepared using natural ingredients and natural derivatives to which *Moringa* extracts were added at 2% and 4% final concentration (Table 6).

The method of emulsion preparation involves the independent preparation of the aqueous phase and the oil phase in different beakers. The aqueous phase is placed on a magnetic stirrer plate; when the temperature reaches 70 °C, proceed with the addition of the xanthan gum until its complete dissolution. Meanwhile, the oily phase is heated up to 70 °C. When both phases are completely dissolved and are at the appropriate temperature, the oily phase is slowly added to the aqueous phase and dispersed by an emulsifier turbo. For this operation, the ULTRA-TURRAX^®^ T18 BASIC emulsifier, Ika Werke GmbH, Germany, was used.

The obtained emulsion is slowly stirred until cooled.

### 4.6. Patch Test

#### 4.6.1. Ethical Requirements

The study was carried out in compliance with the following ethical requirements:

- All of the subjects participating in the study are healthy volunteers at least 18 years old;
- All of the subjects participating in the study are selected under the supervision of a dermatologist according to inclusion/non-inclusion criteria;
- The study participation was on a voluntary basis;
- All of the subjects participating in the study are informed of the aim and the nature of the study;
- All of the subjects participating in the study are informed of the potential risks involved;
- All of the subjects participating in the study give their informed consent signed at the beginning of the study;
- Before volunteers were exposed to the product to be tested, all relevant safety information about the product itself and each ingredient were collected and evaluated;
- All of the study procedures are carried out in accordance with the ethical principles for the medical research (Ethical Principles for Medical Research involving Human Subjects, adopted by the 18th WMA General Assembly Helsinki, Finland, June 1964 and successive amendments);
- All necessary precautions were taken to avoid adverse skin reactions;
- If unexpected/adverse skin reactions occur, the dermatologist supervising the test evaluates the severity of the reaction (and reports it in the data collecting sheet) and if necessary proceeds with the appropriate therapy.

#### 4.6.2. Subjects Selection

Twenty volunteers were recruited to take part in the test in accordance with the following inclusion and non-inclusion criteria:

- Inclusion criteria: male and/or female subjects; subjects between 18 and 70 years old; healthy subjects; subjects informed about test purposes.
- Non-inclusion criteria: Subjects who do not fit the inclusion criteria; pregnant or breastfeeding women; subjects with marks (for example tattoos, scars, burns) in the tested skin region, which might interfere with clinical evaluation; subjects with dermatological problems in the test area; subjects with medication which may affect skin response; subjects undergoing pharmacological treatment (both locally or systemically); subjects with past history for contact dermatitis; positive anamnesis for atopy.

#### 4.6.3. Withdrawal Criteria

Participants were withdrawn if: they did not follow the conditions of the Study information Sheet that they received after the recruitment; they suffered any illness or accident or developed any condition during the study which could affect the outcome of the study; they no longer wished to participate in the study.

#### 4.6.4. Patch Test

The skin area involved in the product application (forearm) was cleaned with a 70% alcoholic solution to make it more sensitive to product application. The product is applied (about 0.1 mL) by using the Finn Chamber, an 8-mm diameter aluminium disk containing a blotting paper disk soaked with the sample to be tested. The Finn Chamber is fixed to the skin with a tape that had already been tested for its safety that ensures the occlusive application of the product (model Curatest^®^ F, adhesive strips for patch test, Lohmann and Rauscher International GMBH and Co., Rengsdorf, Germany).

The applied quantity was sufficient to saturate the pad without overflowing from it when applied to the skin. The product was left in contact with the skin surface for 48 h. The cutaneous reactions are analysed 15 min, 1 h, and 24 h after Finn Chamber removal. A Finn Chamber containing a blotting paper disk soaked with distilled water was applied and used as a negative control.

#### 4.6.5. Clinical Examination and Scoring

Skin reactions are evaluated 15 min, 1 h, and 24 h after patch removal according to the scores reported in Table 7, which describes the severity of erythema, oedema, or other types of skin irritation. 

For each experimental time, the Mean Irritation Index (MII) is then classified following Table 8, which is based on the Mean Irritation Index.

### 4.7. Evaluation of Filtering Parameters

The in vitro Sun Protection Factor determination method used in this work has been recently proposed by us [48] adapting to UVB the ISO 24443:2012 standard for in vitro UVA protection determination.

In vitro SPF spectrophotometric evaluation was performed by measuring absorbance (calculated from transmittance) using a SHIMADZU UV-2600 spectrophotometer provided with an integrating sphere ISR 2600 60 mm and coupled with SPF determination software and a polymethylmethaclylate (PMMA) plate. Approximately 15 µL of glycerin served as reference. We used an in vitro approach that involves applying a thin film of product on an artificial substrate that must be as similar as possible to human skin as concerns its physical characteristics. Via spectrophotometric measures, the amount of UV radiation passing through the film can be evaluated. The substrate used for this study was PMMA plates (WW5 PMMA plates have been purchased from Schonberg GmbH, Munich, Germany), a substrate easily handled and that can be supplied with a 5-µm reproducible roughness with an area of 25 cm^2^ [48].

Furthermore, photostability studies were carried out, as prescribed by the ISO 24443:2012 procedure, with a solar simulator device (Suntest CPSþ; Atlas, Linsengericht, Germany) equipped with a Xenon lamp, an optical filter to cut off wavelengths shorter than 290 nm, and an IR-block filter to avoid thermal effects.

The SPF in vitro is calculated as follows from the spectral absorbance characteristics:

$$In\;vitro\;SPF=\;\frac{\int_{\lambda =290\mathrm{nm}}^{\lambda =400\mathrm{nm}}E\left(\lambda \right)I\left(\lambda \right)d\left(\lambda \right)}{\int_{\lambda =290\mathrm{nm}}^{\lambda =400\mathrm{nm}}E\left(\lambda \right)I\left(\lambda \right)10^{-A\left(\lambda \right)}d\left(\lambda \right)}$$

where *E*(*λ*) is the erythema action spectrum (CIE-1987) at a wavelength *λ*, *I*(*λ*) is the spectral irradiance received from the UV source at a wavelength *λ*, *A*(*λ*) is the monochromatic absorbance of the test product layer at a wavelength *λ*, and *d*(*λ*) is the wavelength step (1 nm).

The UVA protection factor UVAPF_0_ has been calculated for each non-irradiated plate individually:

$${UVAPF}_{0}=\;\frac{\int_{\lambda =320nm}^{\lambda =400nm}P\left(\lambda \right)I\left(\lambda \right)d\left(\lambda \right)}{\int_{\lambda =320nm}^{\lambda =400nm}P\left(\lambda \right)I\left(\lambda \right)10^{-A\left(\lambda \right)C}d\left(\lambda \right)}$$

where *P*(*λ*) = Persistent Pigment Darkening (PPD) action spectrum, *I*(*λ*) = spectral irradiance received from the UV source (UVA 320–400 nm for PPD testing), *A*(*λ*) = Mean monochromatic absorbance of the test product layer, *C* = Coefficient of adjustment, and *dλ* = Wavelength step (1 nm). The Critical Lambda describes the amplitude of the protection across all of the UV spectra (280–400 nm). Moreover, in particular, it is defined as the wavelength at which 90% of the area under the absorbance curve (AUC) is reached starting from 290 nm. The UVA/UVB ratio is defined as the ratio of the mean absorbance from two wavelength ranges (UVA 320–400 nm and UVB 290–320 nm). This value, similarly to the Critical Lambda, provides an evaluation of the amplitude of the protection across the UV spectra without considering the amount of the filtering activity. Values near to 1 are indicative of broad spectrum activity.

### 4.8. Cell Cultures

#### 4.8.1. Colo 38 Cell Line 

The human melanoma Colo 38 cells were cultured in a humidified atmosphere of 5% CO_2_ in RPMI-1640 medium (Lonza, Verviers, Belgium) supplemented with 10% fetal bovine serum (FBS; Biowest, Nuaillé, France), 50 units/mL penicillin (Lonza, Verviers, Belgium), and 50 μg/mL streptomycin (Lonza, Verviers, Belgium).

#### 4.8.2. Cell Proliferation Assays 

Colo 38 cells were seeded (40,000 cells/mL) in 24-well plates in RPMI medium in the presence of 5% FBS. Extracts, decoction, and oil (PB1-PB4) were added in serial dilutions in order to obtain different concentrations (0.05, 0.5, 5, 50, and 500 µg/mL) and incubated for a further 4 days. After 48 and 72 h, cells were harvested, suspended in physiological solution, and counted with a Z2 Coulter Counter (Coulter Electronics, Hialeah, FL, USA) [49]. The cell number/mL was determined as IC_50_ after 2–3 days of culture when the untreated cells are in the log phase of cell growth.

The methanolic extract derived from leaves of *Moringa oleifera* was solubilized in MeOH to prepare stock and working solutions. The hydroalcoholic extract from leaves was solubilized in water. The decoction from leaves was solubilized in water.

### 4.9. Statistical Analysis

Relative standard deviations and statistical significance (Student’s *t*-test; *p* ≤ 0.05) were given where appropriate for all data collected. One-way ANOVA and Least Significant Difference (LSD) post hoc Tukey’s honest significant difference test were used for comparing the bioactive effects of different samples. All computations were made using the statistical software STATISTICA 6.0 (StatSoft Italia s.r.l., Padova, Italy).

## Figures and Tables

**Table 1:** Antioxidant activity of the three different extracts of *Moringa oleifera* leaves. Each value was obtained from three experiments (mean ± SE).

	DPPH (IC_50_) µg/mL	PCL µmol TE/g	FRAP µmol TE/g	ORAC µmol TE/g
Hydroalcoholic extract	232.6 ± 7.61	506.8 ± 3.19	496.6 ± 8.74	2942.8 ± 27.28
Methanolic extract	305.8 ± 12.15	367.1 ± 6.96	418.3 ± 12.24	2272.5 ± 14.72
Water extract	232.8 ± 0.60	512.1 ± 10.30	369.24 ± 27.52	2345.2 ± 10.64

**Table 2:** Total phenol content of leaf extracts of *Moringa oleifera*.

	Total Phenol Content µg GAE/mg
Hydroalcoholic extract	47.7 ± 1.58
Methanolic extract	33.6 ± 1.81
Water extract	51.9 ± 4.88

**Table 3:** Percentages of Rutin, Quercetin, Ellagic acid, Chlorogenic acid, and Ferulic acid in the three different dried extracts of *M. oleifera* leaves. Each value was obtained from three analyses (mean ± SD).

	Percentage of Detected Compounds (w/w)				
	Rutin	Quercetin	Ellagic Acid	Chlorogenic Acid	Ferulic Acid
Hydroalcoholic extract	1.58 ± 0.057	0.26 ± 0.001	0.20 ± 0.007	0.23 ± 0.002	0.16 ± 0.004
Methanolic extract	1.22 ± 0.088	Not detected	0.16 ± 0.001	0.17 ± 0.003	1.04 ± 0.059
Water extract	1.02 ± 0.030	Not detected	0.09 ± 0.004	0.36 ± 0.013	0.55 ± 0.008

**Table 4:** Sun Protection Factor in vitro (mean ± DS) and UVA Protection Factor (mean ± DS), λc (nm) and UVA/UVB Ratio of the tested formulations.

	SPF	UVAPF_0_	λc (nm)	Ratio UVA/UVB
Negative control	1.03 ± 0.01	1.00 ± 0.012	290	0.95
Hydroalcoholic extract 2%	1.51 ± 0.02	1.19 ± 0.01	388	1.00
Hydroalcoholic extract 4%	1.71 ± 0.06	1.35 ± 0.04	385	0.93
Methanolic extract 2%	1.55 ± 0.02	1.21 ± 0.01	389	0.99
Methanolic extract 4%	1.90 ± 0.06	1.50 ± 0.04	387	0.94
Water extract 2%	1.70 ± 0.01	1.19 ± 0.01	373	0.85
Water extract 4%	2.01 ± 0.02	1.44 ± 0.01	381	0.91

**Table 5:** Anti-proliferative effects of *Moringa oleifera* extracts on human melanoma Colo-38 cells.

*Moringa oleifera* Extracts	Anti-Proliferative Effect (IC_50_)
Methanolic extract	na
Hydroalcoholic extract	30.64 ± 2.37 μg/mL
Water extract	na

na = no activity at the used concentrations.

**Table 6:** Oil-in-water (O/W) emulsion formulation scheme.

	Components	%
A.	PHASE I	77–94
1.	Acqua dem.	74–87
2.	Phytic acid (Dermofeel PA3)	0.1–1
3.	Benzyl Alcohol, Glyceryl Caprylate, Benzoic Acid, Propanediol (Kem Nat Beta)	1–1.5
4.	Xanthan gum (Keltrol CG-T)	0.3
5.	*Moringa oleifera* Leaf Extract	2–4
B.	PHASE II	6–23
6.	Glyceryl Stearate Citrate (and) Cetearyl Alcohol (and) Glyceryl Caprylate (Symbiomuls GC)	2–5
7.	Cetearyl alcohol	1–3
8.	Caprylic/capric triglyceride (Myritol 318)	1–5
9.	Coco-caprylate (Cetiol C5)	1–5
10.	Undecane (and) Tridecane (Cetiol ultimate)	1–5

**Table 7:** Scores about erythema, oedema, or other skin irritations.

No erythema	0
Light erythema (hardly visible)	1
Clearly visible erythema	2
Moderate erythema	3
Serious erythema (dark red with possible formation of light scars)	4
No oedema	0
Light oedema (hardly visible)	1
Light oedema	2
Moderate oedema (about 1 mm raised skin)	3
Strong oedema (extended swelling even beyond the application area)	4

**Table 8:** Classification of the medium irritation index (according to the amended Draize classification).

Mean Irritation Index (MII)	
<0.5	Non irritating
From 0.5 to 2.0	Slightly irritating
From 2.0 to 5.0	Moderately irritating
From 5.0 to 8.0	Strongly irritating
